# Development of a UPLC-ESI-MS/MS method for the determination of triamcinolone acetonide in human plasma and evaluation of its bioequivalence after a single intramuscular injection in healthy volunteers

**DOI:** 10.3389/fphar.2023.1223112

**Published:** 2023-07-11

**Authors:** Pengfei Zhao, Ying Qi

**Affiliations:** ^1^ Department of Pharmacology, School of Pharmacy, China Medical University, Shenyang, China; ^2^ Department of Radiology, Shengjing Hospital of China Medical University, Shenyang, China

**Keywords:** triamcinolone acetonide, UPLC, MS, bioequivalence, pharmacokinetics

## Abstract

**Introduction:** Triamcinolone acetonide (TA) is commonly used in the treatment of various inflammatory conditions. To ensure its efficacy and safety, it is important to accurately determine its concentration in human plasma and evaluate its bioequivalence. In this study, an efficient ultra-performance liquid chromatography-electrospray ionization-tandem mass spectrometry (UPLC-ESI-MS/MS) method was developed for the quantification of TA in human plasma after a single intramuscular injection. The internal standard used in this method was cortisone acetate (CA).

**Methods:** TA and CA were extracted from plasma using ethyl acetate and N-hexane (4:1, v/v), separated on a C18 reverse-phase column with a mobile phase of acetonitrile-water containing 1% formic acid (55:45, v/v), and analyzed by UPLC-ESI-MS/MS. Multiple-reaction monitoring was performed using the transitions *m/z* 435.4→397.3 for TA and *m/z* 403.4→163.1 for CA.

**Results:** The developed UPLC-ESI-MS/MS method demonstrated linearity over a concentration range of 0.53–21.20 ng/mL, with a lower limit of quantification of 0.53 ng/mL. The intra- and inter-run precision values ranged from 3.007% to 9.960% and 3.528% to 11.26%, respectively. The intra- and inter-run accuracy ranges were −1.962% to −6.577% and −3.371% to 0.348%, respectively. The matrix effect, extraction recovery, and stability of TA all met the acceptance criteria recommended by the National Medical Products Administration (NMPA) for bioassays. In healthy volunteers who received a single intramuscular injection of 80 mg of either the test or reference formulation of TA, various pharmacokinetic parameters were determined. *C*_max_ was found to be 8.616 ± 1.232 and 8.285 ± 1.218 ng/mL for the test and reference formulations, respectively. *T*_max_ was approximately 1.833 ± 0.243 and 1.861 ± 0.230 h. The *t*_*1/2*_ was calculated to be 181.249 ± 78.585 and 201.782 ± 83.551 h. *AUC*_*0-720*_ was 835.642 ± 297.209 and 830.684 ± 331.168 ng h/mL, *AUC*_*0-∞*_ was 991.859 ± 355.939 and 1018.665 ± 420.769 ng h/mL for the test and reference formulations, respectively. The average relative bioavailability of TA, determined using *AUC*_*0-720*_, was 105.4 ± 26.9%. Bioequivalence was evaluated through variance analysis and a double unilateral test, and the 90% confidence intervals of *AUC*_*0-720*_, *C*_*max*_, and *AUC*_*0-∞*_ were 92.8%–113.4%, 99.1%–109.1%, and 89.7%–110.9%, respectively (all *p* > 0.05).

**Discussion:** These results met the bioequivalence criteria set by the NMPA, indicating that the developed UPLC-ESI-MS/MS method accurately determined TA concentrations in the plasma of healthy Chinese volunteers and that the test and reference formulations exhibited bioequivalence in these individuals.

## Introduction

Triamcinolone acetonide (TA) is a new synthetic fluorine long-term corticosteroid used for the corticosteroid treatment of various diseases. It has a molecular formula of C_24_H_31_FO_6_ and a molecular weight of 434.48. Its white crystalline powder is odorless, soluble in acetone and water, and slightly soluble in chloroform and ethanol.

TA is mainly used to treat allergic diseases, skin diseases, diffuse rheumatoid arthritis, and other connective tissue diseases when the patient is severely debilitated and has failed to respond to conventional drugs. When oral corticosteroids are not feasible, intramuscular injection is significantly effective for these diseases. TA can also be injected intraarticularly or intracapsularly and administered directly to the tendon sheath or joint capsule.

The anti-inflammatory immunity mechanism of TA is to reduce congestion, reduce capillary permeability, inhibit the movement of inflammatory cells (lymphocytes, granulocytes, macrophages, etc.) to the inflammatory site, prevent inflammatory mediators (such as kinins, histamine, and slow reactive substances) from reacting, inhibit phagocytic cell function, stabilize the lysosomal membrane, prevent complements from participating in the inflammatory response, and inhibit the repair of tissue damage after inflammation.

TA has shown good clinical results in treating inflammatory and allergic diseases ([Bielory et al., 2009; Weinstein et al., 2009; Cleary et al., 2010; Leder et al., 2011)]. However, due to its rapid destruction in the liver when administered orally, it is ineffective in this route ([Shin et al., 2000)]. Intramuscular or other methods of administration ([Soma et al., 2011; McLeod et al., 1985)] are necessary to bypass the first-pass effect that may occur with oral administration and ensure proper absorption of the drug. Several high-performance liquid chromatography (HPLC) (Döppenschmitt et al., 1996; Choi et al., 2000; [Shin et al., 2000; Vieira et al., 2010; Amran et al., 2011)], HPLC-mass spectrometry (MS) ([Silva et al., 2009)], gas chromatography (GC)-MS ([Courtheyn et al., 1998)], HPLC-tandem MS (MS/MS) (Taylor et al., 2004; Lim et al., 2006; Qu et al., 2007; De Orsi et al., 2008; [Herrero et al., 2013; Matabosch et al., 2014)], and ultra-performance liquid chromatography (UPLC)-MS/MS (Liu et al., 2015; [Sun et al., 2018; )] methods have been reported for the determination of TA. The most sensitive assay is the UPLC-ESI-MS/MS method with rabbit plasma, which uses a liquid-liquid extraction and has a lower limit of quantification (LLOQ) of 10 pg/mL ([Sun et al., 2018)]. However, there are currently no available studies on the pharmacokinetics and bioequivalence of TA in human plasma after intramuscular injection. Therefore, it is necessary to establish a efficient method to address this issue.

This study presented the successful development and validation of a highly sensitive UPLC-electrospray ionization-(ESI)-MS/MS method for quantifying TA in human plasma using liquid-liquid extraction, with an impressive LLOQ of only 0.53 ng/mL. This method was then used to investigate the pharmacokinetics and bioequivalence of intramuscularly administered TA in healthy Chinese volunteers, yielding valuable data on the behavior of TA in human plasma.

## Materials and methods

### Formulations and subject selection

In this study, the test formulation was TA injection (40 mg/1 mL/ampule, lot no. 221027, expiration date September 2023), provided by Shenyang Everbright Pharmaceutical Co.Co., Ltd., whereas the reference formulation was TA injection (40 mg/1 mL/ampule, lot no. 133, expiration date November 2023), manufactured by Laboratorio Italiano Biochimico Farmaceutico Lisapharma S. p.A. Eighteen healthy male volunteers, ages between 20 and 27 years and weighing between 56 and 82 kg, were selected based on inclusion criteria. Participants signed informed consent forms, underwent comprehensive physical examinations at the First Clinical Hospital of China Medical University before the experiment, and completed blood, urine, liver, and kidney function tests that were all normal. The medical professionals who conducted the experiment closely monitored blood collection times, and two nurses were responsible for blood collection. The study was conducted at the National Institute for Drug Clinical Experiments, First Affiliated Hospital of China Medical University.

### Study design

To explore the bioequivalence of the test and reference formulations of TA after intramuscular injection, an open, randomized, single-dose crossover experiment was conducted with 18 healthy volunteers divided into two groups of 9. Group T received the test formulation before the reference formulation, whereas Group R received the reference formulation first, followed by the test formulation. The administration of each formulation was separated by a 6-week interval. Before injection of either formulation, blood samples were taken from the participants, and at 7:00 a.m., an 80 mg test or reference formulation was administered intramuscularly. Blood samples were taken at regular intervals of 0.5, 1.0, 1.5, 2.0, 3.0, 5.0, 8.0, 12.0, 24.0, 48.0, 96.0, 192.0, 336.0, 504.0, and 720.0 h after administration, with 3– to 5 mL venous blood drawn from the upper limb and stored at −20°°C until analysis. A standard low-fat meal was given 4.5 h after administration. After the first test period was completed, the same procedure was repeated in the second period after a 6-week clean-out period.

### Chemical materials

**FIGURE 1:**
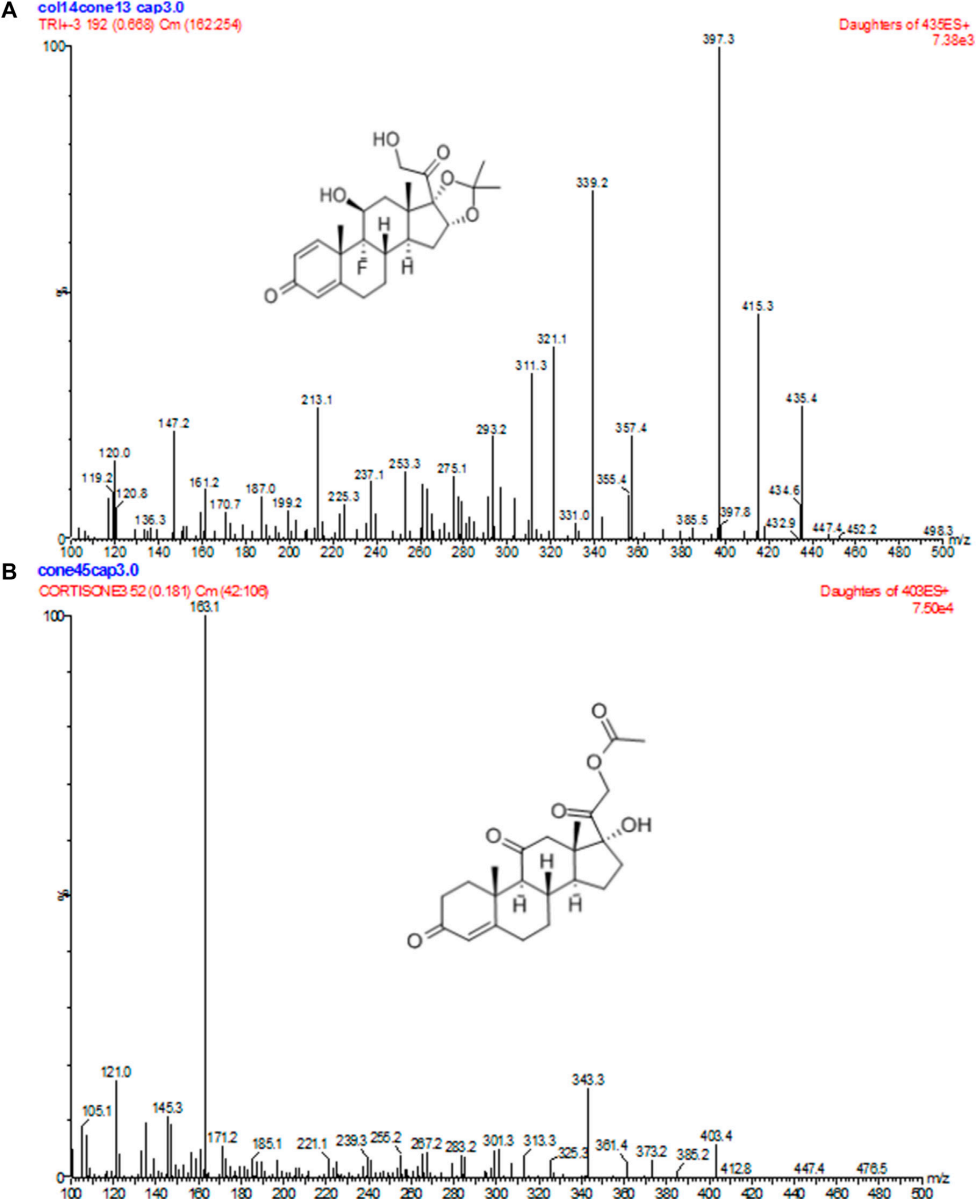
Positive product ion MS of TA **(A)** and CA **(B)** with the [M + H]^+^ ion as a precursor at *m/z* 397 for TA and 163 for CA.

The purity of TA (Figure 1A) and cortisone acetate (CA; Figure 1B) was 99.8% and 99.6%, respectively, which were obtained from the National Institute for the Control of Pharmaceutical and Biological Products (Beijing, China). Ultra-pure water was prepared from Millipore (Bedford, MA, USA), and HPLC-grade acetonitrile was obtained from Merck (Darmstadt, Germany). Analytical-grade ethyl acetate was obtained from Sinopharm Chemical Reagent Co.Co., Ltd. (Shenyang, China). The blank plasma used in the experiment was obtained from the General Hospital of Shenyang Military.

### Instrumentation and conditions

The UPLC-ESI-MS/MS equipment used in the experiment included an ACQUITY™ ultra-performance LC quaternary pump, an ACQUITY™ autosampler, and a Micromass^®^® Quattro Premier XE ES-MS/MS equipped with an ESI ion source (Waters Corp., Milford, MA, USA). Data acquisition and analysis were performed using Masslynx version 4.1. UPLC separation was carried out on an ACQUITY™ BEH C18 column (50 × 2.1 mm, 1.7 μm, Waters Corp.) at 40°°C. The mobile phase was a mixture of acetonitrile/water containing 1% formic acid (55:45, v/v), and a constant mobile phase flow rate of 0.2 mL/min was employed throughout the analysis. The total run time was 2.0 min. Nitrogen was used to assist nebulization during UPLC-ESI-MS/MS, and MS detection was performed using an electrospray ion source in the positive ionization mode. The ion spray for TA was optimized at a spray voltage of 3000 V and a capillary temperature of 350°°C, with the nitrogen sheath, ion sweep, and auxiliary set at 45, 10, and 10 psi, respectively. Collision energies of 13 V were optimized for TA and CA, and multiple-reaction monitoring (MRM) was used for data acquisition. MRM fragmentation transitions were *m/z* 435.4→397.3 for TA and *m/z* 403.4→163.1 for CA, with a scan dwell time of 0.2 s set for each channel.

### Plasma sample processing

In this study, 500 µL plasma was taken, and 50 µL of 40% acetonitrile aqueous solution was added. Then, 50 µL of 500 ng/mL CA internal standard (I.S.) solution comprising of 40% acetonitrile-water mixture was added. The mixture was vortexed for 1 min before adding 3 mL ethyl acetate/hexane (4:1, v/v). The solution was vortexed for 3 min minutes and centrifuged at 4000 rpm for 10 min, then the supernatant 500 µL was taken and transferred to a separate tube. The residue was dried under a stream of nitrogen and dissolved with 100 µL mobile phase. The mixture was vortexed for 1 min at 15,000 rpm. After centrifugation for 3 min, 10 µL supernatant was injected for analysis.

### Method specificity

**FIGURE 2:**
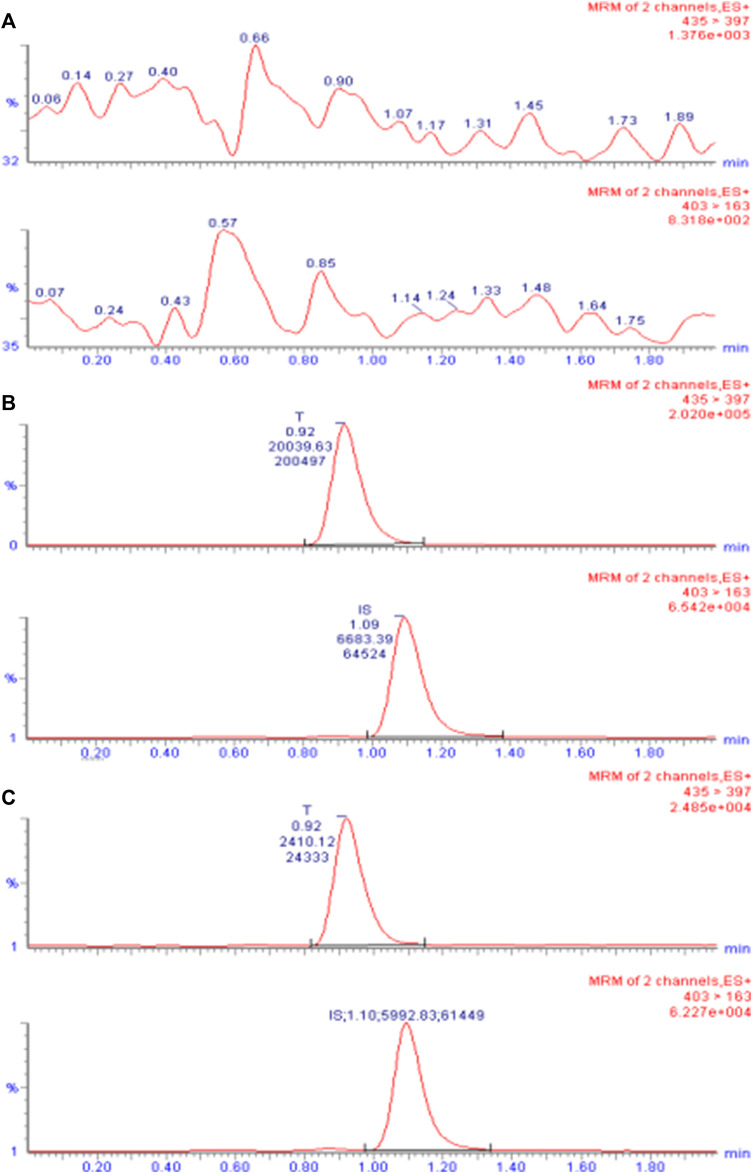
UPLC-ESI-MS/MS chromatogram of TA in plasma. **(A)** Chromatogram of the blank plasma sample. **(B)** Chromatogram of blank plasma spiked with TA (10 ng/mL) and I.S. CA (50 ng/mL). **(C)** Chromatogram of the plasma sample obtained from subject 7 after intramuscular injection of 80 mg reference formulation at 504.0 h.

The primary excimer ions generated by TA and CA under the ESI(+) mode were *m/z* 435.4→397.3 and 403.4→163.1, respectively. During quantitative analysis, they were used as product ions to monitor TA and CA. Blank plasma collected from six different individuals was processed according to the sample processing method, and a 10 µL sample was injected to obtain a typical chromatogram (Figure 2A). A certain concentration of the standard solution and I.S. was spiked into blank plasma, and the chromatogram was obtained using the same method (Figure 2B). Chromatograms were also obtained from plasma samples collected from healthy subjects after intramuscular injection (Figure 2C). The retention times of TA and CA were 0.92 and 1.09 min, respectively. Results indicated that endogenous substances in the blank plasma did not interfere with TA and CA determination.

### Matrix effect

As the UPLC/MS/MS detection method was based on soft ionization MS, the possible dielectric effects in the analysis process were investigated by preparing two samples: one with 500 µL blank plasma and the other with 500 µL deionized purified water. Then, 50 µL low, medium, and high concentrations (1.06, 5.30, and 21.20 ng/mL) of TA solutions were spiked, and three samples were analyzed for each concentration level. A 10 µL sample was injected, and the chromatogram was recorded to obtain the peak area response values of TA and CA. The matrix effect was determined by comparing the mean peak area of the sample prepared with blank plasma to that of the same concentration sample prepared with deionized purified water. The matrix effects were 95.788%, 100.954%, and 101.079% for the three concentrations, respectively, ranging from 85% to 115%. The I.S. matrix effect was 100.554% (Supplementary Table S1).

### Calibration curve and LLOQ

**FIGURE 3:**
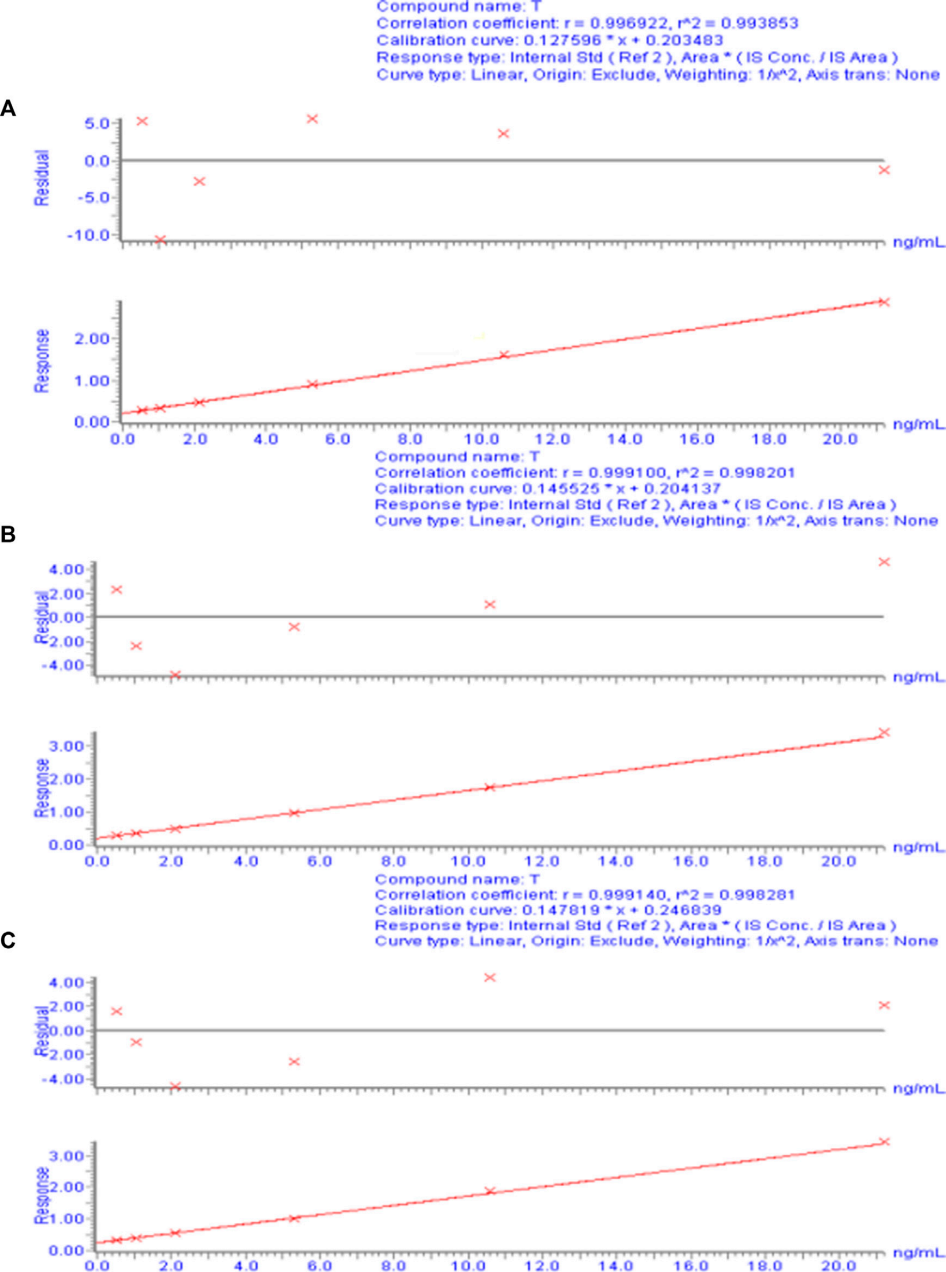
Calibration curve of the plasma drug concentration of TA measured by UPLC-ESI-MS/MS. **(A)** Calibration curve for day 1. **(B)** Calibration curve for day 2. **(C)** Calibration curve for day 3.

For each concentration point of the designed calibration curve, 500 μL blank plasma was taken, and a series of standard solutions were spiked to prepare TA plasma standard series samples corresponding to concentrations of 0.53, 1.06, 2.12, 5.30, 10.60, and 21.20 ng/mL. Following the “Plasma sample processing” method, each concentration was used for single sample analysis, and 10 µL was injected. The chromatogram was recorded, with TA blood concentration as the abscissa and TA and CA peak area ratio as the ordinate. The weighted least-squares method was used for regression analysis, and the obtained linear regression equation was the calibration curve. The results of the 3-day validation method and the calibration curve regression equations for each batch of the methodology are presented in Supplementary Table S2. Figure 3 shows a typical calibration curve. Supplementary Table S1 shows each concentration point of the standard curve. According to the calibration curve, the linear range of TA was 0.53– to 21.20 ng/mL, and LLOQ was 0.53 ng/mL. Supplementary Table S3 shows the accuracy and precision results of LLOQ.

### Precision and accuracy

For each concentration, 500 µL blank plasma was taken, and quality control (QC) samples with low, medium, high and TA concentrations (1.06, 5.30, and 21.20 ng/mL, respectively) were prepared according to the method described in “Calibration curve and LLOQ” and processed according to the “Plasma sample processing” method. Five samples were analyzed for each concentration level, and the continuum determination was performed over 3 days. The measured TA concentration in the QC sample was calculated based on the calibration curve of the same day. The accuracy and precision of this method were calculated based on the measured TA concentration in the QC sample. The intra- and interday precision and accuracy checks are presented in Supplementary Tables S4a, b, respectively.

### Extraction recovery and relative recovery

For each sample, 500 µL blank plasma was taken, and TA QC plasma samples with low, medium, and high concentrations (1.06, 5.30, and 21.20 ng/mL) were prepared according to the method described in “Precision and accuracy” and processed according to the “Plasma sample processing” method. Three samples were analyzed for each concentration level. Another 500 µL blank plasma was taken for each sample and processed according to the “Plasma sample processing” method (without adding 50 µL of 40% acetonitrile aqueous solution). The obtained supernatant was spiked with 50 µL of the corresponding concentration standard solution, vortexed and mixed for 1 min, and blow-dried under a stream of nitrogen, and the residue was dissolved with 100 µL mobile phase. After vortexing for 1 min at 15,000 rpm, the solution was centrifuged for 3 min, and 10 µL supernatant was injected and analyzed to obtain the corresponding peak areas (average of three determinations). Extraction recovery was calculated as the ratio of the peak areas of the two treatments for each concentration. The recovery rates of low, medium, and high concentrations of TA (1.06, 5.30, and 21.20 ng/mL) were 77.406%, 80.110%, and 99.000%, respectively, and the recovery rate of the I.S. was 90.251% (Supplementary Table S5a). Using 500 µL blank plasma for each sample, 1.06, 5.30, and 21.20 ng/mL TA QC plasma samples were prepared according to the “Precision and accuracy” method and processed using the “Plasma sample processing” method. Three samples were analyzed for each concentration level, and the concentration of the QC samples was calculated based on the calibration curve of the day. Relative recovery was calculated as the ratio of the measured concentration to the spiked concentration for each concentration level. The relative recoveries of this method were 112.358%, 103.874%, and 102.898% at low, medium, and high concentrations of TA (1.06, 5.30, and 21.20 ng/mL, respectively; Supplementary Table S5b).

### Sample stability

For each sample, 500 µL blank plasma was used to prepare TA QC plasma samples with low, medium, and high concentrations (1.06, 5.30, and 21.20 ng/mL) as described in “Precision and accuracy,” with three samples per concentration level. QC samples were processed according to “Plasma sample processing” and left at room temperature for 24 h for determination. In Supplementary Table S6a, the treated TA plasma samples were stable for 24 h at room temperature. QC samples were prepared and stored at −20°°C for 120 days, processed separately using the “Plasma sample processing” method, and determined. In Supplementary Table S6b, TA plasma samples stored at −20°°C were stable for 120 days. Furthermore, QC samples were prepared and subjected to three freeze-thaw cycles, with the concentration calculated after each cycle. After processing using the “Plasma sample processing” method, TA plasma samples were stable after three freeze-thaw cycles (Supplementary Table S6c). To evaluate their stability, the TA stock solution (200 ng/mL) and the I.S. stock solution (500 ng/mL) were stored at 4°°C for 30 days. Afterward, 10 µL was injected and analyzed to obtain the corresponding peak areas (Supplementary Table S7). Results showed that TA and I.S. stock solutions were stable within 30 days at 4°°C. Overall, the analytical method met the requirements of relevant regulations and could be used to determine TA plasma concentrations in pharmacokinetic studies.

## Results

### Determination of plasma samples and analysis of results

**FIGURE 4:**
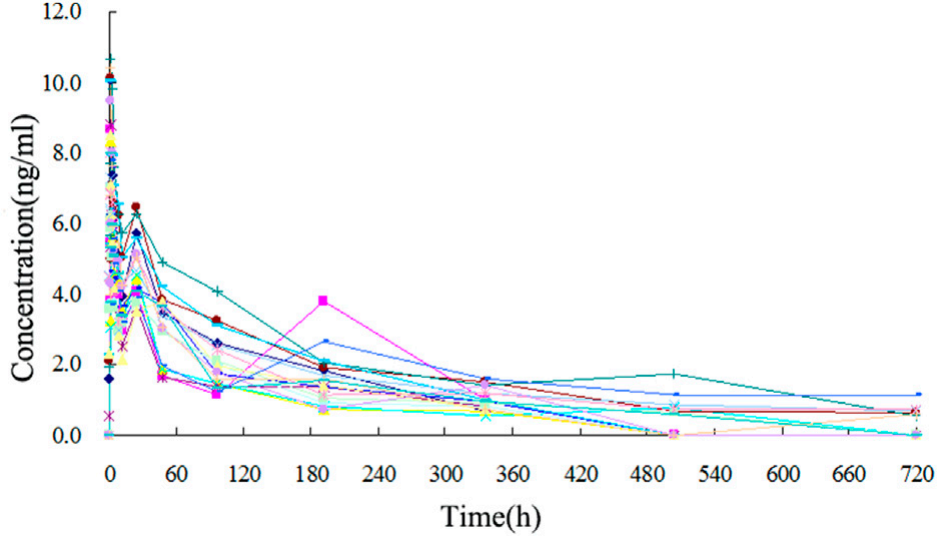
Plasma concentration-time curve of TA after intramuscular injection of 80 mg test formulation in 18 healthy subjects.

**FIGURE 5:**
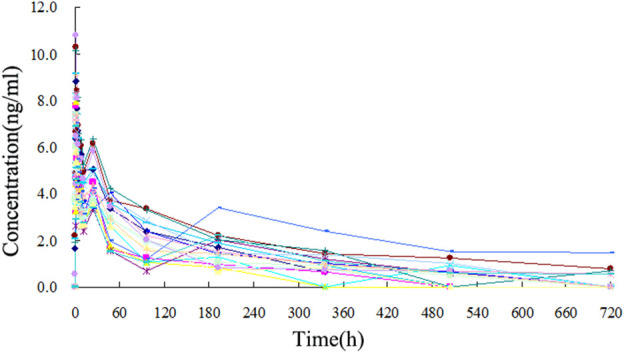
Plasma concentration-time curve of TA after intramuscular injection of 80 mg reference formulation in 18 healthy subjects.

**FIGURE 6:**
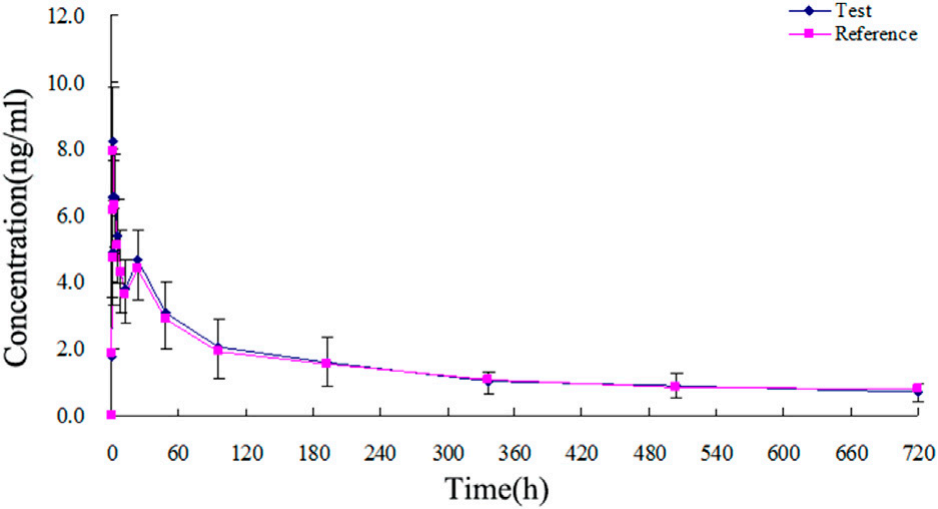
Mean plasma concentration-time curves of test and reference formulations of TA injection after intramuscular injection of 80 mg in 18 healthy subjects.

The TA plasma concentrations in healthy subjects were determined at each time point after intramuscular injection of the reference and test formulations (Supplementary Tables S2, S3, respectively). The corresponding plasma concentration-time curves are displayed in Figures 4, 5. The mean plasma concentration-time curve for all 18 healthy subjects is shown in Figure 6.

### Data processing

The plasma drug concentration-time points of all 18 healthy subjects after intramuscular injection were collected and entered into a Masslynx 4.1 workstation for data analysis. DAS 2.0 was used to calculate the pharmacokinetic parameters and bioequivalence. The trapezoidal method was applied to calculate AUC_0-720_ and AUC_0-∞_ using the concentration-time curves. Furthermore, *t*_1/2_ was calculated from the concentration-time point of the elimination phase using the semilogarithmic mapping method. The measured *T*_max_ and *C*_max_ and the obtained pharmacokinetic parameters are presented in Supplementary Tables 8, 9. The relative bioavailability (Fr) was calculated using the AUC_0-720_ of the test and reference formulations with the formula: Fr = (AUC test formulation / AUC reference formulation) × 100% (Supplementary Table S10).

### Pharmacokinetic cCalculations and bBioequivalence

After intramuscular injection of 80 mg test and reference formulations in healthy subjects, the pharmacokinetic parameters obtained were as follows: *C*_max_ was 8.616 ± 1.232 and 8.285 ± 1.218 ng/mL, *T*_max_ was 1.833 ± 0.243 and 1.861 ± 0.230 h, *t*_1/2_ was 181.249 ± 78.585 and 201.782 ± 83.551 h, AUC_0-720_ was 835.642 ± 297.209 and 830.684 ± 331.168 ng h/mL, and AUC_0-∞_ was 991.859 ± 355.939 and 1018.665 ± 420.769 ng h/mL, respectively. The average relative bioavailability of TA was 105.4% ± 26.9%, as calculated by AUC_0-720_. The 90% confidence interval (CI) of the test formulation AUC_0-720_ was 92.8%– to 113.4% of the corresponding parameters of the reference formulation, and the 90% CI of the test formulation *C*_max_ was 99.1%– to 109.1% of the corresponding parameters of the reference formulation. The 90% CIs of AUC_0-∞_ of the test formulation was 89.7%– to 110.9% of the corresponding parameters of the reference formulation (Supplementary Tables S11–S13). The evaluation of bioequivalence was performed according to the protocol requirements, indicating that if the 90% confidence limit of AUC_0-∞_ and *C*_max_ for the test formulation were within 80%– to 125% and 70%– to 143%, respectively, compared to the reference formulation, the test formulation would be considered bioequivalent. In this study, the test formulation met these criteria ([National Medical Products Administration and Center for Drug Evaluation, 2009)], indicating that it was bioequivalent to the reference formulation.

### Tolerability

No adverse effects were reported for the 18 healthy subjects during the bioequivalence experiment, indicating that both formulations were well tolerated at the doses tested. After the experiment, four subjects (2, 4, 15, and 20) reported cough, hoarseness, runny nose, and other symptoms during follow-up physical examinations. The doctor’s diagnosis concluded that the symptoms were due to influenza, likely unrelated to the experimental drug. The study period coincided with a particularly cold winter season, which may have contributed to the onset of cold symptoms. All subjects fully recovered from the cold symptoms, as confirmed by telephone follow-up after the end of the study. The comprehensive evaluation of clinical examination and doctor’s consultation indicated that the experimental drug did not contribute to the reported symptoms.

## Discussion

In this study, the developed ethyl acetate/hexane (4:1, v/v) liquid-liquid extraction method was superior to other extraction methods. Compared to the different ratios of solvent combinations, such as dichloromethane/methanol, the ethyl acetate/hexane method provides a higher yield and selectivity for TA extraction from human plasma. Its low sample volume requirement and shorter extraction time make it more efficient than conventional methods. Its selectivity and accuracy were compared to other methods published in the literature. They were found to be more successful in terms of recovery of TA from human plasma ([Qu et al., 2007; Lim et al., 2006; Shen et al., 2010)]. The percentage extraction of TA using the ethyl acetate/hexane (4:1, v/v) method was significantly higher than that obtained by other extraction methods ([Taylor et al., 2004; Lim et al., 2006; Matabosch et al., 2014; Liu et al., 2015; Sun et al., 2018; Yao, Q et al., 2020)]. Overall, these results demonstrated the superiority of the ethyl acetate/hexane (4:1, v/v) liquid-liquid extraction method developed in terms of effectiveness, cost-efficiency, and simplicity compared to other methods.

UPLC-ESI-MS/MS is a chromatographic technique that uses MS as detectors. It combines the high separation capability of UPLC with the high sensitivity and selectivity of MS, making it a powerful tool in drug metabolism and pharmacokinetic studies. In this study, UPLC-ESI-MS/MS was used to develop a efficient method to analyze TA in human plasma. This method showed impressive selectivity, sensitivity, and specificity, leading to accurate compound quantification. UPLC-ESI-MS/MS facilitated fast and precise analysis, reducing the time and cost of analysis compared to traditional methods, for example, HPLC, HPLC-MS, HPLC-MS/MS, GC, and GC-MS, among them, the GC related methods also require the analyte to be volatile, this may have a detrimental effect on the analyte and lead to an increase in the detection limit, while also making the sample preparation process more complex and time-consuming. The HPLC or HPLC-MS/MS are inferior to the method used in this study in terms of separation efficiency, sampling volume, sensitivity, throughput, precision, and accuracy. Additionally, although other literature reports used the same method as this study, their tested samples were mostly non-biological or non-human samples. Therefore, this study is the first to apply this method to determine the bioequivalence of TA in human samples. The application of this novel method for evaluating TA bioequivalence after a single intramuscular injection in healthy volunteers demonstrated its ability to accurately determine the pharmacokinetic parameters of the drug. Overall, this study highlighted the importance and uniqueness of UPLC-ESI-MS/MS as an innovative analytical tool in pharmaceutical research and development.

In this study, 18 healthy subjects were randomly cross-injected with 80 mg TA test formulation and 80 mg TA reference formulation. The plasma TA concentration was determined by the UPLC-ESI-MS/MS method. After reviewing the literature, results of pharmacokinetics parameters of TA in healthy people after intramuscular injection measured by UPLC-ESI-MS/MS method were not reported. Although some studies measured the TA metabolite concentration in urine after intramuscular injection ([Matabosch et al., 2014)], there were some differences in pharmacokinetic parameters between humans and animals ([Soma et al., 2011; Liu et al., 2015; Sun et al., 2018; Yao, Q et al., 2020)], which may have been caused by the difference in species. There were also some differences in the pharmacokinetic parameters of healthy people between the dosage forms (Lim et al., 2006; [Qu et al., 2007; Shen et al., 2010)], which may have been caused by different dosage forms. Results of methodological verification showed that the linear range of the calibration curve of TA plasma concentration was 0.53– to 21.20 ng/mL (*r*r^2^ > 0.99), and LLOQ was 0.53 ng/mL. The intra- and interday coefficients of variation and the relative recovery rate of extraction also met the requirements of biological sample analysis. The reliability and accuracy of this test method were confirmed. The efficient assay is suitable for pharmacokinetic studies of TA in human subjects.

The concentration-time curves of the 18 healthy subjects after intramuscular injection of the two formulations showed good fitting relationships (Figures 4–6), suggesting that the two formulations were bioequivalent *in vivo*. The trapezoidal area method estimated the relative bioavailability at 105.2% ± 26.1%. Other pharmacokinetic parameters also demonstrated that the metabolic process and pharmacokinetic parameters of the two formulations in healthy subjects were bioequivalent. Statistical analysis, including three-factor analysis of variance, one-way and two-tailed t-tests, and (1-2α) CI analysis, showed no significant differences in the main pharmacokinetic parameters between the two formulations, indicating that the test and reference formulations were bioequivalent in healthy people.

## Conclusions

Based on the experimental results described above, the test and reference formulations were bioequivalent in healthy Chinese-fasted male volunteers who received a single 80 mg TA injection. Results confirmed that the formulations met the bioequivalence definition of the NMPA regulatory guidelines.

## Data Availability

The original contributions presented in the study are included in the article/Supplementary Material, further inquiries can be directed to the corresponding author.

## References

[B1] Amran M. Khafagy E. S. Leder H. A. Jabs D. A. Galor A. Dunn J. P. (2011). Periocular triamcinolone acetonide injections for cystoid macular edema complicating noninfectious uveitis. Am. J. Ophthalmol. 152, 441–448.e2. 10.1016/j.ajo.2011.02.009 21652023

[B2] Bielory L. Georges G. Gross G. (2009). Treating the ocular symptoms of seasonal allergic rhinitis with triamcinolone acetonide aqueous nasal spray. Ann. Allergy Asthma Immunol. 103, 80–81. 10.1016/S1081-1206(10)60148-2 19663132

[B3] Choi J. S. Shin S. C. Burm J. P. (2000). Circadian changes in the pharmacokinetics of triamcinolone acetonide in rabbits. Res. Commun. Mol. Pathol. Pharmacol. 107, 233–238. 11484877

[B4] Cleary C. A. Lanigan B. O'Keeffe M. (2010). Intracameral triamcinolone acetonide after pediatric cataract surgery. J. Cataract. Refract. Surg. 36, 1676–1681. 10.1016/j.jcrs.2010.04.038 20870112

[B5] Courtheyn D. Vercammen J. Logghe M. Seghers H. De Wasch K. De Brabander H. (1998). Determination of betamethasone and triamcinolone acetonide by GC-NCI-MS in excreta of treated animals and development of a fast oxidation procedure for derivatisation of corticosteroids. Analyst 123, 2409–2414. 10.1039/a804921a 10435270

[B6] De Orsi D. Pellegrini M. Pichini S. Mattioli D. Marchei E. Gagliardi L. (2008). High-performance liquid chromatography-diode array and electrospray-mass spectrometry analysis of non-allowed substances in cosmetic products for preventing hair loss and other hormone-dependent skin diseases. J. Pharm. Biomed. Anal. 48, 641–648. 10.1016/j.jpba.2008.06.008 18656319

[B7] Döppenschmitt S. A. Scheidel B. Harrison F. Surmann J. P. (1996). Simultaneous determination of triamcinolone acetonide and hydrocortisone in human plasma by high-performance liquid chromatography. J. Chromatogr. B Biomed. Appl. 682, 79–88. 10.1016/0378-4347(96)00060-6 8832428

[B8] Herrero P. Borrull F. Marcé R. M. Pocurull E. (2013). Pressurised liquid extraction and ultra-high performance liquid chromatography-tandem mass spectrometry to determine endogenous and synthetic glucocorticoids in sewage sludge. Talanta 103, 186–193. 10.1016/j.talanta.2012.10.030 23200376

[B9] Leder H. A. Jabs D. A. Galor A. Dunn J. P. Thorne J. E. (2011). Periocular triamcinolone acetonide injections for cystoid macular edema complicating noninfectious uveitis. Am. J. Ophthalmol. 152, 441–448.e2. 10.1016/j.ajo.2011.02.009 21652023

[B10] Lim J. G. Shah B. Rohatagi S. Bell A. (2006). Development of a dry powder inhaler, the Ultrahaler, containing triamcinolone acetonide using *in vitro*-*in vivo* relationships. Am. J. Ther. 13, 32–42. 10.1097/01.mjt.0000145357.13410.a9 16428920

[B11] Liu H. Yang M. Wu P. Guan J. Men L. Lin H. (2015). Simultaneous determination of triamcinolone acetonide palmitate and triamcinolone acetonide in beagle dog plasma by UPLC-MS/MS and its application to a long-term pharmacokinetic study of triamcinolone acetonide palmitate lipid emulsion injection. J. Pharm. Biomed. Anal. 104, 105–111. 10.1016/j.jpba.2014.11.028 25497892

[B12] Matabosch X. Pozo O. J. Papaseit E. Farré M. Marcos J. Segura J. (2014). Detection and characterization of triamcinolone acetonide metabolites in human urine by liquid chromatography/tandem mass spectrometry after intramuscular administration. Rapid Commun. Mass Spectrom. 28, 1829–1839. 10.1002/rcm.6965 25559453

[B13] McLeod D. T. Capewell S. J. Law J. MacLaren W. Seaton A. (1985). Intramuscular triamcinolone acetonide in chronic severe asthma. Clin. Trials. 40, 840–845. 10.1136/thx.40.11.840 PMC1020562 3906999

[B14] National Medical Products AdministrationCenter for Drug Evaluation (2009). Guideline for bioavailability and bioequivalence studies of generic drug products. Available at: https://www.nmpa.gov.cn/wwwroot/gsz05106/08.pdf .

[B15] Qu J. Qu Y. Straubinger R. M. (2007). Ultra-sensitive quantification of corticosteroids in plasma samples using selective solid-phase extraction and reversed-phase capillary high-performance liquid chromatography/tandem mass spectrometry. Anal. Chem. 79, 3786–3793. 10.1021/ac062184r 17411010

[B16] Shen L. You Y. Sun S. Chen Y. Qu J. Cheng L. (2010). Intraocular and systemic pharmacokinetics of triamcinolone acetonide after a single 40-mg posterior subtenon application. Ophthalmology 117, 2365–2371. 10.1016/j.ophtha.2010.03.033 20678801

[B17] Shin S. C. Bum J. P. Choi J. S. (2000). Enhanced bioavailability by buccal administration of triamcinolone acetonide from the bioadhesive gels in rabbits. Int. J. Pharm. 209, 37–43. 10.1016/s0378-5173(00)00542-1 11084244

[B18] Silva P. S. Singh R. J. Bakri S. J. Lising R. S. Santiago D. E. Uy H. S. (2009). Vitreous concentration of triamcinolone acetonide after a single transseptal depot injection. Ocul. Immunol. Inflamm. 17, 216–220. 10.1080/09273940802687838 19585367

[B19] Soma L. R. Uboh C. E. You Y. Guan F. Boston R. C. (2011). Pharmacokinetics of intra-articular, intravenous, and intramuscular administration of triamcinolone acetonide and its effect on endogenous plasma hydrocortisone and cortisone concentrations in horses. Am. J. Vet. Res. 72, 1234–1242. 10.2460/ajvr.72.9.1234 21879982

[B20] Sun W. Ho S. Fang X. R. O'Shea T. Liu H. (2018). Simultaneous determination of triamcinolone hexacetonide and triamcinolone acetonide in rabbit plasma using a highly sensitive and selective UPLC-MS/MS method. J. Pharm. Biomed. Anal. 153, 267–273. 10.1016/j.jpba.2018.02.052 29550043

[B21] Taylor R. L. Grebe S. K. Singh R. J. (2004). Quantitative, highly sensitive liquid chromatography-tandem mass spectrometry method for detection of synthetic corticosteroids. Clin. Chem. 50, 2345–2352. 10.1373/clinchem.2004.033605 15486026

[B22] Vieira M. L. Singh R. P. Derendorf H. (2010). Simultaneous HPLC analysis of triamcinolone acetonide and budesonide in microdialysate and rat plasma: Application to a pharmacokinetic study. J. Chromatogr. B Anal. Technol. Biomed. Life Sci. 878, 2967–2973. 10.1016/j.jchromb.2010.08.048 20884303

[B23] Weinstein S. Qaqundah P. Georges G. Nayak A. (2009). Efficacy and safety of triamcinolone acetonide aqueous nasal spray in children aged 2 to 5 years with perennial allergic rhinitis: A randomized, double-blind, placebo-controlled study with an open-label extension. Ann. Allergy Asthma Immunol. 102, 339–347. 10.1016/S1081-1206(10)60340-7 19441606

[B24] Yao Q. Guo Y. Xue J. Kong D. Li J. Tian X. (2020). Development and validation of a LC-MS/MS method for simultaneous determination of six glucocorticoids and its application to a pharmacokinetic study in nude mice. J. Pharm. Biomed. Anal. 179, 112980. 10.1016/j.jpba.2019.112980 31744668

