# Supplementary material for: Development of a UPLC-ESI-MS/MS method for the determination of triamcinolone acetonide in human plasma and evaluation of its bioequivalence after a single intramuscular injection in healthy volunteers
Source: Front Pharmacol. 2023 Jul 11;14:1223112. doi: 10.3389/fphar.2023.1223112 (PMC10367002; doi:10.3389/fphar.2023.1223112)
Supplement: Supplementary file 1 [file Table3.DOCX]

**Table 1 Matrix effect of the UPLC-ESI-MS/MS method for TA determination in plasma samples (n = 3)**

| Plasma Concentration | Ratio of peak area response values of blank plasma  to samples prepared with deionized purified water | | | |
| --- | --- | --- | --- | --- |
|  | TA（ng/ml） | | | CA（ng/ml） |
|  | 1.06 | 5.30 | 21.20 | 50 |
| Matrix Effect（%） | 98.990 | 100.750 | 104.478 | 97.728 |
|  | 94.682 | 99.882 | 102.030 | 99.484 |
|  | 93.661 | 102.229 | 96.730 | 104.450 |
| Mean（%） | 95.778 | 100.954 | 101.079 | 100.554 |
| SD | 2.829 | 1.187 | 3.961 | 3.446 |
| RSD（%） | 2.953 | 1.176 | 3.918 | 3.427 |

**Table 2 Calibration curve parameters for TA determination in plasma samples by UPLC-ESI-MS/MS.**

| Sample Batch | Slope | Intercept | *r*^2^ |
| --- | --- | --- | --- |
| 1 | 0.127596 | 0.203483 | 0.9969 |
| 2 | 0.145525 | 0.204137 | 0.9991 |
| 3 | 0.147819 | 0.246839 | 0.9991 |

**Table 3 Accuracy and precision of LLOQ of TA plasma samples (n = 5)**

| Sample Number | concentration（0.53 ng/ml） |
| --- | --- |
| 1 | 0.531 |
| 2 | 0.499 |
| 3 | 0.528 |
| 4 | 0.430 |
| 5 | 0.511 |
| Mean | 0.500 |
| SD | 0.041 |
| RSD（%） | 8.229 |
| RE（%） | -5.698 |

**Table 4a Intraday precision and accuracy of TA plasma samples (n = 5)**

| Sample Batch | concentration（ng/ml） | | |
| --- | --- | --- | --- |
|  | 1.06 | 5.30 | 21.20 |
| 1 | 1.077 | 4.643 | 21.377 |
|  | 0.940 | 5.383 | 21.407 |
|  | 1.195 | 4.728 | 20.842 |
|  | 0.972 | 4.794 | 20.084 |
|  | 0.989 | 5.209 | 20.210 |
| Mean（ng/ml） | 1.035 | 4.951 | 20.784 |
| SD | 0.103 | 0.325 | 0.625 |
| RE（%） | -2.396 | -6.577 | -1.962 |
| RSD（%） | 9.960 | 6.563 | 3.007 |

**Table 4b Interday precision and accuracy of TA plasma samples (n = 5)**

| Sample Batch | concentration（ng/ml） | | |
| --- | --- | --- | --- |
|  | 1.06 | 5.30 | 21.20 |
| 1 | 0.906 | 5.392 | 21.083 |
|  | 1.149 | 4.808 | 21.024 |
|  | 1.102 | 4.719 | 21.445 |
|  | 0.915 | 4.688 | 21.977 |
|  | 0.970 | 4.923 | 22.461 |
| 2 | 1.212 | 5.988 | 22.772 |
|  | 1.186 | 6.053 | 21.516 |
|  | 0.930 | 5.961 | 21.453 |
|  | 1.188 | 5.954 | 21.293 |
|  | 0.938 | 5.882 | 21.536 |
| 3 | 0.952 | 4.660 | 20.207 |
|  | 0.951 | 4.706 | 20.423 |
|  | 0.990 | 4.658 | 20.509 |
|  | 1.009 | 4.919 | 21.123 |
|  | 0.966 | 4.775 | 20.284 |
| Mean（ng/ml） | 1.024 | 5.206 | 21.274 |
| SD | 0.110 | 0.586 | 0.751 |
| RE（%） | -3.371 | -1.779 | 0.348 |
| RSD（%） | 10.771 | 11.260 | 3.528 |

**Table 5a Extraction recoveries of TA and CA in plasma (n = 3)**

| **Plasma**  **Concentration** | TA Concentration（ng/ml） | | | CA Concentration（ng/ml） |
| --- | --- | --- | --- | --- |
|  | 1.06 | 5.30 | 21.20 | 50 |
| **Extraction Recoveries**  （%） | 86.252 | 89.413 | 100.012 | 83.520 |
|  | 79.740 | 82.575 | 97.359 | 89.746 |
|  | 66.225 | 68.343 | 99.629 | 97.488 |
| Mean（%） | 77.406 | 80.110 | 99.000 | 90.251 |
| SD | 10.215 | 10.749 | 1.434 | 10.147 |
| RSD（%） | 13.197 | 13.418 | 1.448 | 11.243 |

**Table 5b Relative recoveries of TA (n = 3)**

| **Plasma**  **Concentration** | TA (ng/ml) | | |
| --- | --- | --- | --- |
|  | 1.06 | 5.30 | 21.20 |
| **Relative Recoveries**（%） | 107.453 | 102.283 | 103.509 |
|  | 114.057 | 105.132 | 102.991 |
|  | 115.566 | 104.208 | 102.193 |
| Mean（%） | 112.358 | 103.874 | 102.898 |
| SD | 4.315 | 1.453 | 0.663 |
| RSD（%） | 3.840 | 1.399 | 0.644 |

**Table 6a Stability of standard plasma samples of TA after 24 h of treatment (n = 3)**

| Storage Time（h） | TA Concentration（ng/ml） | | |
| --- | --- | --- | --- |
|  | 1.06 | 5.30 | 21.20 |
| 0 | 0.955 | 5.994 | 22.446 |
|  | 1.074 | 5.605 | 23.446 |
|  | 1.146 | 5.849 | 21.044 |
| 6 | 1.203 | 5.884 | 21.697 |
|  | 1.297 | 5.799 | 21.132 |
|  | 1.257 | 5.792 | 20.840 |
| 12 | 1.348 | 5.249 | 20.313 |
|  | 1.119 | 5.089 | 20.286 |
|  | 1.102 | 6.058 | 20.647 |
| 24 | 0.999 | 5.312 | 20.921 |
|  | 0.981 | 5.260 | 20.227 |
|  | 0.929 | 5.184 | 21.104 |
| Mean （ng/ml） | 1.167 | 5.702 | 21.317 |
| SD | 0.122 | 0.331 | 1.049 |
| RSD（%） | 10.480 | 5.799 | 4.922 |

**Table 6b Stability of TA plasma samples stored at -20℃ for 120 days (n = 3)**

| Storage Time (day) | TA Concentration（ng/ml） | | |
| --- | --- | --- | --- |
|  | 1.06 | 5.30 | 21.20 |
| 0 | 1.027 | 5.561 | 23.869 |
|  | 1.160 | 6.004 | 24.024 |
|  | 1.080 | 5.622 | 23.545 |
| 30 | 1.237 | 6.158 | 23.302 |
|  | 1.267 | 6.208 | 22.696 |
|  | 1.256 | 6.120 | 21.662 |
| 120 | 1.118 | 5.561 | 22.527 |
|  | 1.277 | 5.459 | 22.557 |
|  | 1.098 | 5.581 | 22.431 |
| Mean （ng/ml） | 1.169 | 5.808 | 22.957 |
| SD | 0.093 | 0.306 | 0.775 |
| RSD（%） | 7.961 | 5.265 | 3.375 |

**Table 6c Stability investigation of TA samples after three freeze-thaw cycles (n = 3)**

| freeze-thaw cycles（times） | TA Concentration（ng/ml） | | |
| --- | --- | --- | --- |
|  | 1.06 | 5.30 | 21.20 |
| 0 | 1.079 | 5.548 | 21.421 |
|  | 1.155 | 5.484 | 21.572 |
|  | 1.136 | 5.476 | 22.000 |
| 1 | 1.216 | 5.295 | 21.610 |
|  | 1.030 | 5.391 | 21.043 |
|  | 1.136 | 5.673 | 21.223 |
| 2 | 1.347 | 5.767 | 22.306 |
|  | 1.259 | 5.742 | 21.722 |
|  | 1.288 | 5.668 | 22.827 |
| 3 | 1.079 | 5.399 | 20.142 |
|  | 1.072 | 5.522 | 20.857 |
|  | 1.156 | 5.214 | 21.880 |
| Mean (ng/ml) | 1.163 | 5.515 | 21.550 |
| SD | 0.097 | 0.174 | 0.700 |
| RSD(%) | 8.329 | 3.164 | 3.248 |

**Table 7 Stability of TA and CA stock solution stored at 4℃ for 30 days (n = 3)**

| Storage Time (day) | Peak area of TA stock solution (200ng/ml) | Peak area of CA stock solution(500ng/ml) |
| --- | --- | --- |
| 0 | 188180.828 | 201346.016 |
|  | 188849.328 | 202779.781 |
|  | 188868.375 | 198021.594 |
| 1 | 169751.438 | 191013.469 |
|  | 169865.172 | 186410.328 |
|  | 172480.078 | 184689.313 |
| 30 | 200853.188 | 215270.625 |
|  | 204315.172 | 218025.516 |
|  | 211103.828 | 196461.672 |
| Mean (ng/ml) | 188251.934 | 199335.368 |
| SD | 15284.236 | 11618.748 |
| RSD（%） | 8.119 | 5.829 |

**Table 8 Pharmacokinetic parameters of the test formulation of TA injection after intramuscular injection of 80 mg in 18 healthy subjects**

| Subject  Number | *t*_1/2_ (h) | *T*_max_ (h) | *C*_max_(ng/ml) | AUC_0-720_ (ng·h·ml^-1^) | AUC_0-∞_ (ng·h·ml^-1^) |
| --- | --- | --- | --- | --- | --- |
| 2 | 103.208 | 2 | 10.076 | 779.182 | 884.065 |
| 3 | 159.936 | 2 | 8.661 | 818.840 | 1116.958 |
| 4 | 106.922 | 2 | 8.330 | 467.729 | 513.833 |
| 5 | 162.380 | 2 | 7.286 | 579.835 | 642.047 |
| 6 | 145.770 | 2 | 8.806 | 515.755 | 680.702 |
| 8 | 325.901 | 2 | 10.144 | 1263.010 | 1565.790 |
| 9 | 269.240 | 2 | 10.657 | 1563.673 | 1768.478 |
| 10 | 149.568 | 1.5 | 7.605 | 644.501 | 856.441 |
| 11 | 140.979 | 2 | 10.025 | 908.839 | 1117.211 |
| 12 | 84.997 | 1.5 | 7.059 | 552.678 | 587.408 |
| 13 | 230.972 | 1.5 | 6.999 | 717.502 | 918.856 |
| 14 | 135.423 | 1.5 | 8.526 | 626.474 | 764.445 |
| 15 | 328.267 | 1.5 | 7.268 | 1092.775 | 1347.843 |
| 16 | 237.303 | 1.5 | 8.007 | 1022.324 | 1155.905 |
| 17 | 69.037 | 2 | 9.474 | 608.525 | 625.014 |
| 18 | 137.990 | 2 | 10.404 | 902.842 | 924.169 |
| 19 | 255.599 | 2 | 7.803 | 1210.915 | 1435.635 |
| 20 | 218.992 | 2 | 7.963 | 766.160 | 948.654 |
| Mean | 181.249 | 1.833 | 8.616 | 835.642 | 991.859 |
| SD | 78.585 | 0.243 | 1.232 | 297.209 | 355.939 |

**Table 9 Pharmacokinetic parameters of 18 healthy subjects after intramuscular injection of 80 mg TA injection reference formulation**

| Subject  Number | *t*_1/2_ (h) | *T*_max_ (h) | *C*_max_(ng/ml) | AUC_0-720_(ng·h·ml^-1^) | AUC_0-∞_ (ng·h·ml^-1^) |
| --- | --- | --- | --- | --- | --- |
| 2 | 105.175 | 2 | 8.813 | 733.771 | 831.986 |
| 3 | 269.687 | 2 | 7.753 | 461.271 | 719.711 |
| 4 | 62.910 | 2 | 7.933 | 312.169 | 345.466 |
| 5 | 255.540 | 2 | 8.177 | 716.675 | 1048.850 |
| 6 | 176.940 | 2 | 6.793 | 698.675 | 852.303 |
| 8 | 295.607 | 2 | 10.293 | 1404.351 | 1733.621 |
| 9 | 325.855 | 2 | 10.161 | 1393.466 | 1709.047 |
| 10 | 270.528 | 1.5 | 8.072 | 841.376 | 1096.475 |
| 11 | 145.766 | 2 | 9.149 | 805.715 | 994.306 |
| 12 | 86.867 | 1.5 | 7.170 | 545.176 | 581.781 |
| 13 | 210.103 | 1.5 | 7.401 | 698.780 | 858.049 |
| 14 | 75.790 | 2 | 7.080 | 485.108 | 505.752 |
| 15 | 266.919 | 1.5 | 8.310 | 982.926 | 1350.936 |
| 16 | 260.351 | 1.5 | 8.216 | 961.944 | 1110.063 |
| 17 | 138.669 | 2 | 10.784 | 746.546 | 808.732 |
| 18 | 251.574 | 2 | 9.115 | 759.229 | 981.553 |
| 19 | 272.283 | 2 | 7.038 | 1538.131 | 1887.888 |
| 20 | 161.508 | 2 | 6.877 | 867.000 | 919.458 |
| Mean | 201.782 | 1.861 | 8.285 | 830.684 | 1018.665 |
| SD | 83.551 | 0.230 | 1.218 | 331.168 | 420.769 |

**Table 10 Calculation results of the relative bioavailability of test and reference formulations for TA injection after intramuscular injection of 80 mg each in 18 healthy subjects**

| Subject  Number | AUC_0-720_ (ng·h·ml^-1^) | | F（%） |
| --- | --- | --- | --- |
|  | test formulation | reference formulation |  |
| 2 | 779.2 | 733.8 | 106.2 |
| 3 | 818.8 | 461.3 | 177.5 |
| 4 | 467.7 | 312.2 | 149.8 |
| 5 | 579.8 | 716.7 | 80.9 |
| 6 | 515.8 | 698.7 | 73.8 |
| 8 | 1263.0 | 1404.4 | 89.9 |
| 9 | 1563.7 | 1393.5 | 112.2 |
| 10 | 644.5 | 841.4 | 76.6 |
| 11 | 908.8 | 805.7 | 112.8 |
| 12 | 552.7 | 545.2 | 101.4 |
| 13 | 717.5 | 698.8 | 102.7 |
| 14 | 626.5 | 485.1 | 129.1 |
| 15 | 1092.8 | 982.9 | 111.2 |
| 16 | 1022.3 | 961.9 | 106.3 |
| 17 | 608.5 | 746.5 | 81.5 |
| 18 | 902.8 | 759.2 | 118.9 |
| 19 | 1210.9 | 1538.1 | 78.7 |
| 20 | 766.2 | 867.0 | 88.4 |
| Mean | 835.6 | 830.7 | 105.4 |
| SD | 297.2 | 331.2 | 26.9 |

**Table 11 ANOVA results of the main pharmacokinetic parameters after intramuscular injection of 80 mg each of the test and reference formulations of TA injection in 18 healthy subjects**

| parameters | F | | |
| --- | --- | --- | --- |
|  | Inter-formulations | Inter-periods | inter-individual |
| lnAUC_0-720_ | 0.193 | 0.094 | 8.337* |
| lnAUC_0-∞_ | 0.002 | 0.000 | 8.498* |
| ln*C*_max_ | 2.057 | 0.356 | 4.973* |

F_0.05_(1,16) = 4.49; F_0.05_(17,16) = 2.29; **p* < 0.05.

**Table 12 Results of the double unilateral test and (1-2α) CI method after intramuscular injection of the test and reference formulations 80 mg each in 18 healthy subjects**

| parameters | *t*_L_ | *t*_H_ | 90%CI_s_ |
| --- | --- | --- | --- |
| lnAUC_0-720_ | 4.320 | 3.442 | 92.8%~113.4% |
| lnAUC_0-∞_ | 3.643 | 3.731 | 89.7%~110.9% |
| ln*C*_max_ | 14.406 | 11.574 | 99.1%~109.1% |

T_(1-0.05)_ (16) = 1.75.

**Table 13 Results of the Wilcoxon test after intramuscular injection of TA with the test and reference formulations 80 mg each in 18 healthy subjects**

|  | Group R | Group T | P | Conclusion |
| --- | --- | --- | --- | --- |
| Mean±SD | 1.86±0.23 | 1.83±0.24 | >0.05 | Qualified |
| Max-Min | 2.00-1.50 | 2.00-1.50 |  |  |
| Median | 2 | 2 |  |  |
