# Supplementary material for: Development of a UPLC-ESI-MS/MS method for the determination of triamcinolone acetonide in human plasma and evaluation of its bioequivalence after a single intramuscular injection in healthy volunteers
Source: Front Pharmacol. 2023 Jul 11;14:1223112. doi: 10.3389/fphar.2023.1223112 (PMC10367002; doi:10.3389/fphar.2023.1223112)
Supplement: Supplementary file 2 [file Table4.DOCX]

Supplementary tables

**Supplementary Table 1 Calibration curve data for TA determination in plasma samples by UPLC-ESI-MS/MS.**

| Sample Batch | concentration（ng/ml） | | | | | |
| --- | --- | --- | --- | --- | --- | --- |
|  | 0.53 | 1.06 | 2.12 | 5.30 | 10.60 | 21.20 |
| 1 | 0.558 | 0.947 | 2.061 | 5.602 | 10.998 | 20.925 |
| 2 | 0.542 | 1.034 | 2.018 | 5.256 | 10.716 | 22.184 |
| 3 | 0.539 | 1.050 | 2.023 | 5.163 | 11.068 | 21.646 |
| Mean（ng/ml） | 0.544 | 1.013 | 2.067 | 5.335 | 10.819 | 21.531 |
| SD | 0.011 | 0.056 | 0.099 | 0.301 | 0.228 | 1.003 |
| RSD（%） | 1.965 | 5.563 | 4.813 | 5.639 | 2.106 | 4.659 |
| RE（%） | 2.604 | -4.396 | -2.519 | 0.668 | 2.062 | 1.563 |

**Supplementary Table 2 Plasma concentration of TA in 18 healthy subjects at different time points after intramuscular injection of the test formulation (T group)**

| Time after injection (h) | | Plasma concentration of TA at each time point after intramuscular injection (ng/ml) | | | | | | | | | | | | | | | |
| --- | --- | --- | --- | --- | --- | --- | --- | --- | --- | --- | --- | --- | --- | --- | --- | --- | --- |
|  |  | 0 | 0.5 | 1.0 | 1.5 | 2.0 | 3.0 | 5.0 | 8.0 | 12.0 | 24.0 | 48.0 | 96.0 | 192.0 | 336.0 | 504.0 | 720.0 |
| Subject  Number | 2 | 0.000 | 1.605 | 4.402 | 6.257 | 10.076 | 7.391 | 6.547 | 5.095 | 3.961 | 5.737 | 3.467 | 2.614 | 1.845 | 0.706 | n.d. | n.d. |
|  | 3 | 0.000 | 0.000 | 3.87 | 5.446 | 8.661 | 5.888 | 6.246 | 4 | 2.971 | 4.058 | 1.707 | 1.155 | 3.782 | 1.014 | n.d. | n.d. |
|  | 4 | 0.000 | 0.000 | 3.255 | 5.433 | 8.33 | 5.569 | 4.602 | 4.298 | 3.513 | 4.439 | 1.882 | 1.475 | 0.75 | 0.687 | n.d. | n.d. |
|  | 5 | 0.000 | 0.000 | 3.088 | 4.498 | 7.286 | 5.5 | 5.803 | 4.513 | 3.231 | 4.534 | 1.894 | 1.483 | 0.829 | 0.546 | 0.764 | n.d. |
|  | 6 | 0.000 | 0.546 | 3.625 | 5.337 | 8.806 | 6.24 | 5.154 | 3.222 | 2.532 | 3.641 | 1.668 | 1.363 | 1.391 | 0.833 | n.d. | n.d. |
|  | 8 | 0.000 | 2.084 | 4.989 | 7.041 | 10.144 | 7.95 | 6.552 | 6.221 | 5.052 | 6.449 | 3.84 | 3.269 | 1.931 | 1.508 | 0.689 | 0.638 |
|  | 9 | 0.000 | 1.925 | 5.676 | 7.698 | 10.657 | 9.797 | 7.593 | 6.274 | 5.743 | 6.295 | 4.9 | 4.094 | 2.071 | 1.4 | 1.76 | 0.529 |
|  | 10 | 0.000 | 4.626 | 6.285 | 7.605 | 7.062 | 5.842 | 4.451 | 3.432 | 3.645 | 4.16 | 3.741 | 1.722 | 1.372 | 0.982 | n.d. | n.d. |
|  | 11 | 0.000 | 2.281 | 5.383 | 7.121 | 10.025 | 7.936 | 7.045 | 6.561 | 5.059 | 5.574 | 4.203 | 3.113 | 2.098 | 1.02 | n.d. | n.d. |
|  | 12 | 0.000 | 3.743 | 6.021 | 7.059 | 5.876 | 5.203 | 3.802 | 2.853 | 3.145 | 3.754 | 2.956 | 2.015 | 0.915 | 0.813 | n.d. | n.d. |
|  | 13 | 0.000 | 3.595 | 5.844 | 6.999 | 6.07 | 5.037 | 3.628 | 2.987 | 3.152 | 3.737 | 2.945 | 2.112 | 1.068 | 0.937 | 0.628 | n.d. |
|  | 14 | 0.000 | 2.352 | 7.077 | 8.526 | 6.28 | 5.463 | 4.177 | 2.817 | 2.141 | 3.537 | 3.798 | 1.991 | 1.431 | 0.718 | n.d. | n.d. |
|  | 15 | 0.000 | 4.52 | 6.303 | 7.268 | 6.402 | 6.262 | 4.878 | 2.981 | 4.336 | 4.691 | 3.376 | 2.549 | 1.672 | 1.172 | 0.863 | 0.681 |
|  | 16 | 0.000 | 4.472 | 6.806 | 8.007 | 6.862 | 6.575 | 4.89 | 3.193 | 4.124 | 5.038 | 3.641 | 2.407 | 1.137 | 1.207 | 0.73 | 0.753 |
|  | 17 | 0.000 | 0.000 | 4.324 | 5.99 | 9.474 | 7.996 | 5.217 | 4.966 | 4.216 | 5.144 | 3.045 | 1.787 | 0.71 | 1.43 | n.d. | n.d. |
|  | 18 | 0.000 | 0.000 | 3.985 | 6.764 | 10.404 | 7.655 | 5.892 | 5.461 | 4.278 | 5.061 | 3.055 | 1.604 | 1.598 | 0.719 | n.d. | 0.578 |
|  | 19 | 0.000 | 0.000 | 3.696 | 5.225 | 7.803 | 5.53 | 4.846 | 4.076 | 3.198 | 3.903 | 1.95 | 1.246 | 2.642 | 1.618 | 1.128 | 1.141 |
|  | 20 | 0.000 | 0.000 | 3.742 | 5.324 | 7.963 | 5.959 | 5.079 | 4.586 | 3.394 | 4.084 | 3.655 | 1.347 | 1.555 | 0.978 | 0.579 | n.d. |
|  | Mean | 0.000 | 2.886 | 4.910 | 6.533 | 8.232 | 6.544 | 5.356 | 4.308 | 3.761 | 4.658 | 3.096 | 2.075 | 1.600 | 1.016 | 0.893 | 0.720 |
|  | SD | 0.000 | 1.371 | 1.286 | 1.134 | 1.622 | 1.290 | 1.112 | 1.243 | 0.926 | 0.906 | 0.940 | 0.802 | 0.755 | 0.313 | 0.389 | 0.221 |

n.d.:The concentration was below the lower limit quantification, and no quantitative tests were performed

**Supplementary Table 3 Plasma concentrations of TA at different time points after intramuscular injection of reference formulation (group R) in 18 healthy subjects**

| Time after injection (h) | | Plasma concentration of TA at each time point after intramuscular injection (ng/ml) | | | | | | | | | | | | | | | |
| --- | --- | --- | --- | --- | --- | --- | --- | --- | --- | --- | --- | --- | --- | --- | --- | --- | --- |
|  |  | 0 | 0.5 | 1.0 | 1.5 | 2.0 | 3.0 | 5.0 | 8.0 | 12.0 | 24.0 | 48.0 | 96.0 | 192.0 | 336.0 | 504.0 | 720.0 |
| Subject  Number  . | 2 | 0.000 | 1.664 | 4.565 | 6.353 | 8.813 | 7.67 | 6.709 | 5.711 | 4.997 | 5.044 | 3.345 | 2.403 | 1.683 | 0.645 | n.d. | n.d. |
|  | 3 | 0.000 | 0.000 | 3.245 | 4.817 | 7.753 | 5.498 | 5.473 | 4.215 | 2.9 | 4.492 | 1.615 | 1.233 | 0.959 | 0.665 | n.d. | n.d. |
|  | 4 | 0.000 | 0.000 | 3.248 | 4.465 | 7.933 | 5.397 | 4.219 | 4.24 | 2.718 | 3.736 | 1.733 | 1.072 | 0.823 | n.d. | n.d. | n.d. |
|  | 5 | 0.000 | 0.000 | 3.456 | 5.307 | 8.177 | 6.108 | 5.99 | 4.509 | 3.004 | 3.917 | 2.602 | 1.068 | 1.267 | n.d. | 0.909 | n.d. |
|  | 6 | 0.000 | 0.000 | 2.629 | 3.902 | 6.793 | 4.684 | 3.968 | 3.242 | 2.392 | 3.366 | 1.547 | 0.671 | 2.02 | 1.187 | 0.596 | n.d. |
|  | 8 | 0.000 | 2.207 | 4.877 | 6.66 | 10.293 | 8.436 | 6.93 | 6.088 | 4.919 | 6.164 | 3.708 | 3.36 | 2.199 | 1.433 | 1.254 | 0.783 |
|  | 9 | 0.000 | 1.932 | 5.353 | 6.092 | 10.161 | 8.151 | 6.564 | 6.276 | 5.222 | 6.323 | 4.251 | 3.317 | 2.037 | 1.547 | n.d. | 0.668 |
|  | 10 | 0.000 | 4.292 | 6.416 | 8.072 | 6.439 | 5.643 | 4.276 | 3.564 | 3.684 | 3.356 | 4.039 | 2.413 | 1.447 | 1.014 | 0.651 | n.d. |
|  | 11 | 0.000 | 2.086 | 4.771 | 6.411 | 9.149 | 7.399 | 6.412 | 5.583 | 4.447 | 5.069 | 3.591 | 2.748 | 1.89 | 0.9 | n.d. | n.d. |
|  | 12 | 0.000 | 4.03 | 6.054 | 7.17 | 5.75 | 5.063 | 3.62 | 2.924 | 2.98 | 3.803 | 2.942 | 1.948 | 0.927 | 0.773 | n.d. | n.d. |
|  | 13 | 0.000 | 3.751 | 6.013 | 7.401 | 6.014 | 5.184 | 3.834 | 2.992 | 3.238 | 3.689 | 2.954 | 2.023 | 1.061 | 0.91 | 0.527 | n.d. |
|  | 14 | 0.000 | 3.431 | 5.395 | 5.841 | 7.08 | 4.904 | 3.717 | 2.684 | 2.711 | 3.553 | 2.625 | 1.7 | 0.704 | 0.825 | n.d. | n.d. |
|  | 15 | 0.000 | 4.883 | 6.964 | 8.31 | 7.387 | 6.652 | 5.05 | 3.415 | 4.355 | 4.866 | 3.831 | 2.838 | 1.412 | 1.378 | 0.996 | n.d. |
|  | 16 | 0.000 | 4.764 | 7.259 | 8.216 | 7.059 | 6.285 | 4.173 | 3.155 | 3.92 | 4.028 | 3.587 | 2.186 | 1.504 | 0.946 | 0.712 | 0.56 |
|  | 17 | 0.000 | 0.564 | 4.391 | 6.474 | 10.784 | 8.106 | 6.115 | 5.515 | 4.642 | 5.843 | 3.463 | 2.041 | 0.813 | 0.739 | 0.705 | n.d. |
|  | 18 | 0.000 | 0.000 | 3.957 | 5.651 | 9.115 | 7.391 | 5.526 | 5.052 | 3.786 | 4.376 | 2.906 | 1.571 | 1.44 | 0.982 | 0.61 | n.d. |
|  | 19 | 0.000 | 0.000 | 3.709 | 5.156 | 7.038 | 5.46 | 4.913 | 4.111 | 3.114 | 4.216 | 1.983 | 1.19 | 3.404 | 2.38 | 1.539 | 1.477 |
|  | 20 | 0.000 | 0.000 | 2.874 | 4.611 | 6.877 | 5.087 | 4.447 | 3.638 | 2.76 | 3.388 | 1.52 | 1.08 | 2.22 | 1.122 | 0.58 | 0.533 |
|  | Mean | 0.000 | 3.055 | 4.732 | 6.162 | 7.923 | 6.284 | 5.108 | 4.273 | 3.655 | 4.402 | 2.902 | 1.937 | 1.545 | 1.090 | 0.825 | 0.804 |
|  | SD | 0.000 | 1.429 | 1.417 | 1.324 | 1.497 | 1.262 | 1.130 | 1.170 | 0.911 | 0.958 | 0.903 | 0.805 | 0.677 | 0.437 | 0.322 | 0.389 |

n.d.:The concentration was below the lower limit quantification, and no quantitative tests were performed
